# Feasibility and acceptability of autism adapted safety plans: an external pilot randomised controlled trial

**DOI:** 10.1016/j.eclinm.2024.102662

**Published:** 2024-06-01

**Authors:** Jacqui Rodgers, Sarah Cassidy, Mirabel Pelton, Jane Goodwin, Janelle Wagnild, Nawaraj Bhattarai, Isabel Gordon, Colin Wilson, Phil Heslop, Emmanuel Ogundimu, Rory C. O’Connor, Sheena E. Ramsay, Ellen Townsend, Luke Vale

**Affiliations:** a Population Health Sciences Institute, Newcastle University, NE1 7RU, UK; b School of Psychology, University of Nottingham, NG7 2RD, UK; c Autism Research Centre, University of Cambridge, UK; d Department of Anthropology, Durham University, DH1 3LE, UK; e Health Economics Group, Population Health Science Institute, Newcastle University, NE1 7RU, UK; f Social Work, Education and Community Wellbeing, Northumbria University, NE7 7XA, UK; g Department of Mathematical Sciences, Durham University, DH1 3LE, UK; h Suicidal Behaviour Research Lab, School of Health & Wellbeing, University of Glasgow, G12 8TB, UK

**Keywords:** Autism, Autistic adults, Suicide, Self-harm, Safety planning

## Abstract

**Background:**

Autistic people are a high-risk group for self-harm and suicide. There are no evidence-based suicide prevention interventions developed specifically for autistic people. We undertook a pilot feasibility randomised controlled trial of autism adapted safety plans (AASP) to reduce self-harm and suicide for autistic people.

**Methods:**

This study took place in the United Kingdom and followed a randomised, two-arm, controlled design. Autistic adults (n = 53, mean age = 39, gender = 49% female, 29% not male or female) were recruited via third sector organisations and self-referral between 11.8.21 and 19.10.22. Participants were randomised without stratification to usual care with or without AASP. The AASP was completed by the autistic adults together with someone trained to support them. Research staff who completed follow-up assessments were blind to participant allocation. Primary outcomes were feasibility and acceptability. Participants were assessed at baseline, 1 and 6 months. Primary data were analysed under the intention to treat principle. Study protocol is published. The trial is closed to new participants. This study is registered with the ISRCTN registry, ISRCTN70594445.

**Findings:**

53 participants consented, 49 were randomised to either AASP with usual care (n = 25) or usual care (n = 24). 68% of participants in the AASP arm were satisfied with the AASP and 41% rated it as useable. Feedback on the AASP and research methods were positive with suggested adaptations to some outcome measures. Retention and completion of outcomes measures in both arms was excellent, as was fidelity of delivery of the AASP.

**Interpretation:**

Study progression criteria were met, suggesting that the parameters of a future definitive trial of clinical and cost effectiveness of AASP to reduce self-harm and suicide in autistic adults are achievable, with minor recommended adaptions to outcome measures and AASP. Future research should explore the use of AASP in routine clinical practice.

**Funding:**

This study is funded by the NIHR [10.13039/501100001921Public Health Research Programme (NIHR129196)].

**Research in context**

**Evidence before this study**

We searched OVID (Psychinfo, Embase and Medline databases) for articles published from database inception to 16 October 2023, with the terms “ASC” or “ASD” or “Asperg∗” or “Autis∗” or “high functioning” or “pervasive developmental disorder∗” or “PDD” or “HFA and “safety plan∗” and “suicid∗” or “suicide plans” or “suicide attempts” or “attempted suicide” or “parasuicide” “self-harm” or “self-inj∗” without language restrictions. We identified no previous pilot or definitive trials of suicide safety plans for autistic adults. One empirical study reported that clinicians use safety plans with similar frequency for autistic and non-autistic adults but report less confidence in using them to support autistic adults. One commentary drew together existing evidence in support of safety planning as an intervention that has the potential to be tailored to be effective for autistic adults. This commentary concludes that an evidence-based trial is warranted. Other papers echo these findings in children and youth clinical settings though these studies are small, draw on practitioner case studies and call for larger, formal studies.

**Added value of this study**

This pilot RCT provided important feasibility and acceptability data regarding the use of Autism Adapted Safety Plans and describing achievable parameters for a future definitive trial to test the clinical and cost-effectiveness of the AASP to reduce suicide and self-harm. Our findings are the first to show that autistic adults currently experiencing self-harm and suicidal behaviour can be successfully and safely supported to develop an AASP with the potential to reduce rates of self-harm and suicidal behaviour. We found high rates of retention within the study, and overall reports of feasibility and acceptability of research processes.

**Implications of all the available evidence**

The AASP is a promising intervention to reduce the incidence of self-harm and suicide risk amongst autistic adults. A full-scale randomised controlled trial is required to ascertain definitive clinical and cost effectiveness. With minor adaptations the AASP and study processes can inform the delivery of a definitive trial.

## Introduction

Autistic people are at high risk of suicide.^1^ In the UK, autism prevalence is estimated at 1–2% of the general population but a recent psychological autopsy study reported evidence of possible autism amongst 41% of people who died by suicide.^2^ There are currently no evidence-based supports which have been specifically developed for autistic people who experience self-harm or suicidal thoughts and behaviours, even though these experiences are more commonly reported by autistic than non-autistic people.^3^ Accessing mainstream mental health services is often reported to contribute to, rather than alleviate, distress for autistic people.^4^ This reflects growing recognition that the way we measure, conceptualise and understand suicide and self-harm for the general population does not describe the experiences of autistic people.^5^ An international priority setting exercise has highlighted the importance of developing effective tailored clinical tools for suicide prevention for autistic people,^6^ which has been reiterated in the new UK suicide prevention strategy 2023–2028.^7^ Overall, this suggests an urgent need for data to inform the clinical effectiveness of tailored suicide prevention approaches for autistic people. This study is the first step towards achieving this ultimate aim.

Suicide safety plans are a recommended intervention for a range of clinical groups including autistic adults.^8^ Suicide safety plans are a series of hierarchical steps to be followed to help people to stay safe during periods of acute crisis that have demonstrated effectiveness in reducing self-harm and suicidal behaviour amongst non-autistic people.^9^^,^^10^ They can be delivered by a range of professionals and can be adapted to meet the heterogeneous presentations of autistic people, including sensory and communication preferences or areas of passionate interest.^8^ One previous study in the United States reported that clinicians were less confident in using safety plans with autistic clients, but, as yet there is no evidence to inform how to adapt and use safety plans with autistic people.^11^ Our work developed Autism Adapted Safety Plans (adapted from safety plans developed by Stanley & Brown), in partnership with autistic adults and those who support them.^12^^,^^13^ However, no research has yet tested the feasibility and acceptability of AASP, in a randomised controlled trial.

The primary aim was to establish the feasibility and acceptability of the AASP and inform the parameters of a definitive randomised controlled trial. Measuring outcomes of a mental health or suicide intervention trial for autistic people represents a significant challenge given evidence that standardised questionnaires designed to capture the experiences of non-autistic people often lead to spurious results for autistic people.^14^^,^^15^ Thus, secondary aims of this study are to explore the extent to which clinical and health economics outcomes can be accurately measured and reported in a future definitive trial.

## Methods

### Study design

This is an external pilot randomised control trial of a suicide prevention intervention tool aimed at mitigating the risk of self-harm and suicidal behaviour in autistic adults. Participants were assessed at baseline and 1 and 6-month follow-up. The study was carried out in the United Kingdom with participants from diverse locations. Ethical approval was obtained from the National Health Service (NHS) Health Research Authority and Wales Research Ethics Committee (Wales REC 5; REC Reference: 20/WA/0101; IRAS Project ID: 280742). As outlined in the protocol,^12^ stage 1 included Patient Involvement (PPI) focus groups to inform adaptations to the safety plan for autistic people and stage 2 was a single arm feasibility study. Progression criteria were met to inform this stage 3 pilot study randomised controlled trial.

### Participants

Autistic adults with experience of self-harm, suicidal thoughts and behaviours were recruited via non-NHS services (charities, higher education and third sector organisations) and via self-referral route following social media announcements. Inclusion criteria were: (i) a formal diagnosis of autism; (ii) accessing services via social care or third sector organisation or self-referred into the study; (iii) self-reported self-harm, suicidal thoughts or behaviours within the last 6 months; (iv) sufficient English language fluency to complete the safety plan; and (v) aged over 18. Insufficient English language fluency and current psychotic symptoms were exclusion criteria. Participants self-reported their sex/gender using a free text option and provided written informed consent to take part. 32.1% of the sample self-reported having completed a safety plan at some point. However, no participants reported using safety plans currently.

### Randomisation and masking

Participants completed baseline assessments (see Table 1) and were then randomised to receive either the AASP in addition to usual care or usual care only, on a 1 to 1 basis without stratification. Randomisation was done via Sealed Envelope (https://www.sealedenvelope.com) facilitated by an unblinded researcher who informed participants of their randomisation status. The unblinded researcher did not undertake follow up assessments or data analysis.

**Table 1:** Baseline characteristics.

Characteristic	AASP + usual care (n = 25)	Usual care (n = 24)	Overall (n = 49)
**Age (years)**^a^	39 (13)	38 (14)	39 (13)
**Age at diagnosis of autism (years)**^a^	32 (15)	30 (15)	31 (15)
**Gender**			
Female	10 (40%)	14 (58%)	24 (49%)
Male	7 (28%)	4 (17%)	11 (22%)
Other^b^	8 (32%)	6 (25%)	14 (29%)
**Ethnicity**			
White	24 (96%)	22 (92%)	46 (94%)
**Household income**			
Less than £15,599	7 (28%)	4 (17%)	11 (22%)
£15,600 to £36,399	10 (40%)	5 (21%)	15 (31%)
£36,400 or more	3 (12%)	8 (33%)	11 (22%)
Unknown/prefer not to say	5 (20%)	7 (29%)	12 (25%)
**Highest education qualification**			
A-levels or below	7 (28%)	9 (38%)	16 (33%)
University	13 (52%)	8 (33%)	21 (43%)
Postgraduate	5 (20%)	7 (29%)	12 (25%)
**Baseline suicidality**			
Suicidality – primary diagnosis (MINI)	4 (17%)	4 (17%)	8 (17%)
Current suicidality	22 (96%)	20 (83%)	42 (89%)
**Primary psychiatric diagnosis**^c^^,^^d^^,^^e^			
Generalised anxiety disorder	8 (35%)	6 (25%)	14 (30%)
Posttraumatic stress disorder	4 (17%)	5 (21%)	9 (19%)
Unable to answer/not applicable	3 (13%)	1 (4%)	4 (9%)

a Data are mean (SD); all other data are n (%).

b Includes non-binary, agender, asexual, demi-sexual, gender fluid, mostly female, sort of female, trans, trans masculine, and none.

c Refers to current psychiatric diagnosis unless otherwise specified.

d MINI questionnaire in AASP arm is n = 23 due to two missing observations.

e Cells where n > 4 are removed from table to protect participant anonymity.

### Procedure

Interested individuals completed an expression of interest form granting permission for the research team to make contact to provide more information. Individuals who self-referred were either linked to a support worker from a partner organisation or completed the AASP with a researcher. Data consent, data collection and completion of the safety plan took place via telephone or video call to meet the access preference of the participant. Training for support workers and researchers was co-designed with autistic people and included information about suicide and self-harm in autistic people, adaptations from standard safety planning, considerations when working with autistic people, helpful insight into autism, such as the double empathy problem (where both autistic and non-autistic people struggle to understand and empathise with one another),^16^ opportunities to discuss and practise the AASP. With consent AASP completion was audio recorded to determine fidelity. Follow up assessments were undertaken at 1 and 6 months with a full list of measures administered at each time point detailed below and available in the study protocol.^12^ All participants completed a wellbeing plan with a researcher prior to being randomised.^17^ This included information about a trusted person to contact if the research team were worried about a participants wellbeing. The study also utilised a standard operating procedure for categorising and reporting SAE and ESI according to funder guidelines, as detailed in the study protocol.^12^

### The AASP

The original Stanley & Brown safety plan consists of six sections, involving the identification of: (1) warning signs; (2) internal coping strategies; (3) social contacts and locations; (4) family members or friends that may offer help; (5) professionals or agencies to help and (6) how to keep the environment safe.^9^ Adaptations from Stage 1 PPI included clarifying the template to meet autistic thinking and communication styles, inserting “what is important to me” in place of “reasons for living”. The AASP was accompanied by an optional resource pack, which included tools to identify emotions, scales, pictorial representations, and support services to support the autistic person. This resource pack was developed in collaboration with autistic people, their families, and those who support them through PPI focus groups in stage one, and feasibility interviews in stage two of the study.^13^

### Outcomes

Primary outcomes were feasibility and acceptability of the research methods and the AASP to inform a future definitive trial. How feasibility and acceptability would be judged were defined a priori in the study protocol^12^: we assessed acceptability of outcome measures, intervention materials, any perceived benefits of the AASP, whilst feasibility assessed experience of recruitment and randomisation. Specifically, to inform a definitive trial, we recorded: (i) number of autistic people who completed the AASP; (ii) response rates for completion of outcome measures, follow up rates, response rates for questionnaires; (iii) time needed to collect and analyse data; (iv) feedback from participants and service providers on methods of recruitment, randomisation, proposed outcome measures, possible use of reinforcement activities, research procedures and data collection methods; (iv) data from participants and service providers about what comprises treatment as usual. Progression criteria for a definitive trial were: (i) number of participants who completed assessments; (ii) the percentage of participants who rate the usability of the Safety Plans (SPs) on the System Usability Scale^18^ as 68 or above; (iii) the percentage of participants who report satisfaction with the AASP intervention (indicated as a score >20 on the Client Satisfaction Questionnaire-8^19^; and (iv) fidelity of delivery of the AASP manual using a bespoke fidelity checklist (see Supplementary File S1).

We collected data on a number of secondary outcomes to assess their acceptability and usefulness to be able to measure clinical and cost effectiveness of a definitive trial. Secondary outcomes included: the Self-injurious Thoughts and Behaviours Inventory (SITBI)^20^ which records the number of incidents of self-harm and suicidal thoughts and behaviours in a 6-month period. We also assessed suicidal thoughts and behaviours using the Suicidal Behaviours Questionnaire – Autism Spectrum Condition^21^ and a measure of life disadvantage using the Vulnerabilities Experience Quotient (VEQ).^22^ We evaluated the feasibility of health economics measures for a future definitive trial by determining the acceptability (clarity, ease of use) and completeness of bespoke questionnaires about: (i) healthcare resource utilisation; and (ii) time and travel related to healthcare. Data regarding health-related quality of life as a health outcome measure were collected using the EQ-5D-5L.^23^ The Mini-International Neuropsychiatric Interview (MINI)^24^ was administered at baseline to characterise the sample.

### Choice of primary outcome

Our primary aim was to establish the feasibility of undertaking a definitive randomised controlled trial of the AASP to determine its effectiveness. To determine this, we measured multiple parameters using the: (i) System Usability Scale (SUS)^18^ and Client Satisfaction Questionnaire-8 (CSQ-8)^19^ (administered in the AASP + arm only); and (ii) bespoke acceptability and feasibility semi-structured interview for autistic adults and support workers (see Supplementary File S2). The SUS is quick, easy to administer quantitative and can reliably differentiate between useable and unusable systems in small samples.^18^ The CSQ-8 assesses satisfaction with care and has high internal consistency and good validity in mental health outpatient settings.^19^

### Statistical analysis

An overview of the statistical analysis plan is included in the protocol,^12^ published before the analysis. The statistical analysis plan and health economics analysis plan were signed off prior to beginning the analysis. The pilot trial was not designed to estimate a target difference in relative effectiveness but to address outcomes to estimate the parameters for a future trial.^25^^,^^26^ However, due to difficulties recruiting during the COVID-19 pandemic, the final sample was 53. As this was a pilot trial, the reported confidence intervals are for guidance only. All analyses followed the intention-to-treat principle. Measures of usability (SUS) and satisfaction (CSQ-8) were summarized using descriptive statistics in the AASP arm only. Analysis of secondary outcomes, including SITBI, SBQ-ASC, and VEQ, were analysed using logistic regression (binary outcomes) or general linear models (continuous outcomes). All models were adjusted for baseline values, age, and gender; ethnicity was intended to be included as a covariate but due to small numbers across groups this was omitted. To inform a randomisation scheme for a future definitive trial, adaptive LASSO regression models were fit to examine associations between multiple demographic characteristics (household income, educational attainment, employment status, housing situation, physical health, and service access) and SITBI outcomes, with adjustment for allocation and baseline values. Demographics whose coefficients were not forced to zero were considered potentially important. This method has been recently used to identify predictors of non-suicidal self-injury.^27^ Acceptability of health economics outcome measures was analysed using descriptive statistics (number and percentages of questionnaires and missing items). Time needed to administer these tools was summarised from interviewer/participant notes. Safety was analysed in line with the safety reporting criteria in the protocol. Only Serious Adverse Events (SAEs) were captured for participants, none were expected for participants, so all SAEs were classed as unexpected. SAEs were tabulated in frequency tables per trial arms, action taken, outcome and causality in the opinion of the investigator. Events of special interest (ESI) e.g., participant no longer attending work/college, relationship breakdown, housing/financial changes, mental health decline - were summarised per trial arm using frequency tables. Analyses were undertaken in R and Excel. This study is registered with the ISRCTN registry, ISRCTN70594445.

An independent rater (IG) was randomly allocated 20% of recorded AASP sessions to rate for fidelity, using a checklist with a three-point scale (see Supplementary File S1). An additional independent rater (JR) met with IG to ensure inter-rater reliability using the fidelity checklist.

### Role of the funding source

This study was funded by National Institute for Health Research (grant number: PHR NIHR129196). The funders had no role in study design, data collection, data analysis, data interpretation or writing of this report.

## Results

**Fig. 1:**
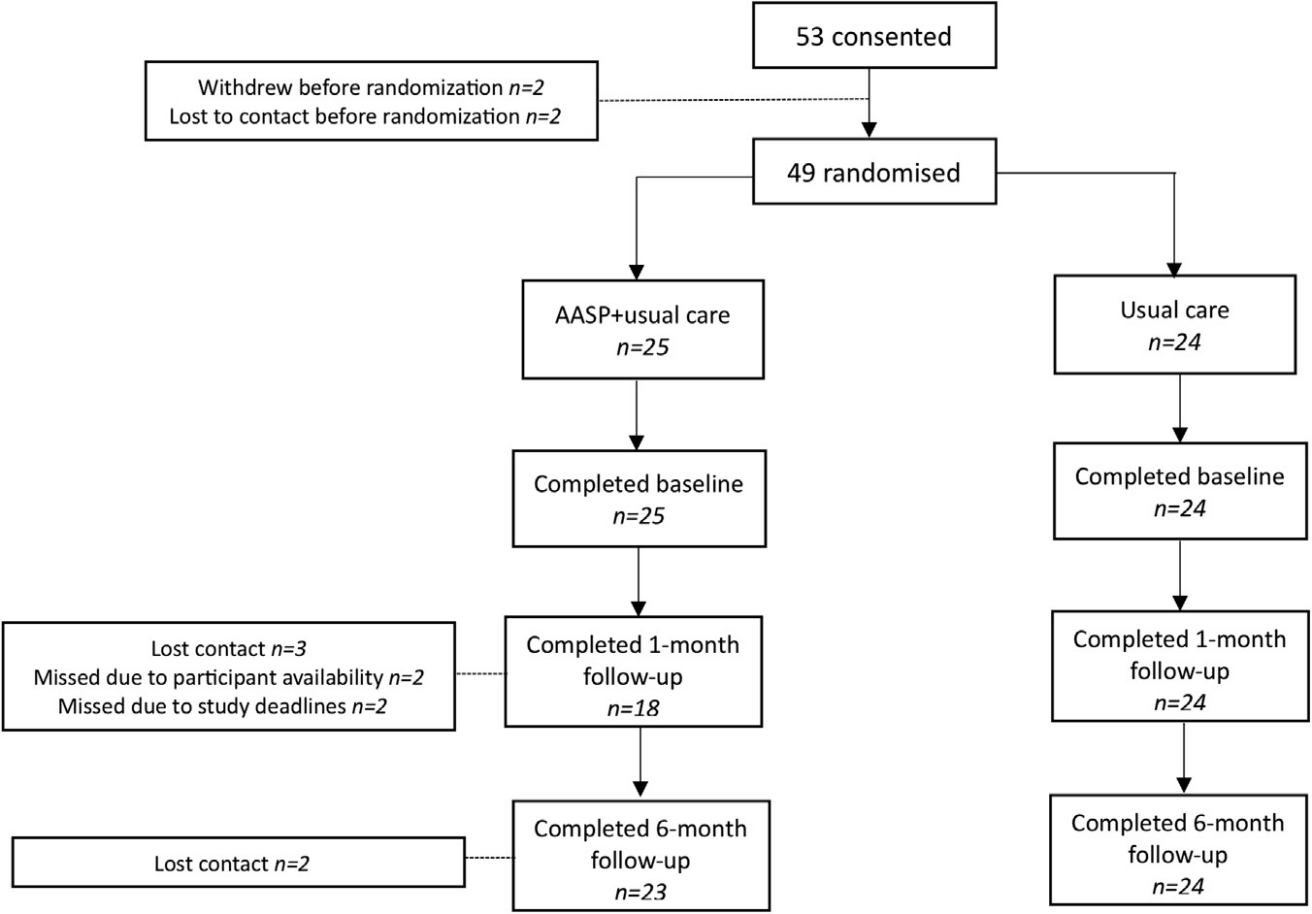
Trial profile.

Recruitment took place between 11.8.21 and 19.10.22. The first participant was consented 11/8/21 and the last participant was consented 19/10/22. The last participant completed their final follow up assessment 25/4/23. The database was locked on 20/06/23 and the SAP was first approved 17/05/23. Of the 53 participants consented, four were not randomised due to withdrawal (n = 2) or lost contact (n = 2). Forty-nine participants were randomised to either AASP + usual care (n = 25) or usual care (n = 24). One participant in the AASP arm was lost to contact after randomisation; all participants were retained in the usual care arm. In the AASP arm, seven and two participants did not complete any assessments at 1 and 6 months, respectively (see Fig. 1).

Participant characteristics are summarised in Table 1. The mean age of participants was 39 years (range 18–70) and mean age at diagnosis of autism was 31 years (range 2–58 years). Approximately half the sample identified as female, and 29% identified with a gender other than man or woman. In both trial arms, the most common mental health diagnosis at baseline based on the MINI questionnaire was generalised anxiety disorder, followed by post-traumatic stress disorder and current suicidality (Table 1).

All retained participants randomised to the AASP completed a safety plan. Completion of the outcomes measures was high. Retention of those randomised was excellent to six-month follow-up (95%). Twenty-two participants in the AASP completed the SUS. Scores on the SUS can range from 0 to 100, with higher scores indicating better usability. The mean score was 61.2 (SD 20.5; range of 19–96). Nine respondents (41%) reported a score of 68 or higher, indicating satisfaction with usability. Satisfaction with the AASP was assessed using the CSQ-8. Of the 19 AASP participants who completed the CSQ-8, the mean score was 24.8 (SD 6.5; range 10–32. Thirteen respondents (68%) indicated they were satisfied with the intervention based on a CSQ-8 score greater than 20.

Descriptive statistics and analyses of the SITBI questionnaire are shown in Supplementary File S3 (Table 1, Table 2). Participants were asked to indicate whether they had ever experienced each of the thoughts/behaviours ever in their life (baseline) or in the past month (at one and six months). Except for gestures, the vast majority of respondents in both arms had reported experiencing each of the thoughts/behaviours at baseline (Supplementary File S3, Supplementary Table S1). These proportions were smaller at one- and six-month follow-up, although a large proportion in both arms (>20%, sometimes up to 96%) reported NA/unable to answer across domains, except for attempts at six months where >85% answered yes or no.

**Table 2:** Parameter estimates between sociodemographic characteristics and SITBI outcomes at six months using logistic regression and adaptive LASSO.

	Ideation		Plan		Thoughts of injury		Non-suicidal self-injury		Any behaviour	
	Logistic regression	Adaptive LASSO	Logistic regression	Adaptive LASSO	Logistic regression	Adaptive LASSO	Logistic regression	Adaptive LASSO	Logistic regression	Adaptive LASSO
**Income**										
Less than £15,600	..	..	..	..	..	..	..	..	..	..
£15,600 to £36,399	0.12	–	0.73	–	−0.23	–	1.23	–	0.37	–
≥£36,400	0.16	–	−0.65	–	2.12	–	3.00	2.58	1.15	–
Unknown/prefer not to say	−1.10	−1.41	0.44	–	0.30	–	1.07	..	0.54	–
**Highest education**										
A-levels or below	..	–	..	..	..	..	..	..	..	..
University	0.27	–	0.89	–	−2.15	–	−0.18	–	0.37	–
Postgraduate	−1.01	−1.40	−1.12	–	−2.15	–	−0.99	−0.86	−1.16	−1.01
**Employment**										
Full time										
Self-employed	−1.04	–	−1.63	−0.66	−2.27	–	−2.07	−1.43	−2.15	−0.91
Unable to work	0.50	–	−0.96	–	−1.29	–	−1.41	–	−0.52	–
Other	0.07	–	−2.68	−0.90	−2.45	–	−4.35	−3.24	−1.40	–
**Housing**										
Lives alone	..	–	..	..	..	..	..	..	..	..
Lives with others	0.82	0.44	−0.36	–	0. .9	–	0.67	–	0.20	–
**Physical health**										
No conditions	..	–	..	..	..	..	..	..	..	..
Conditions	2.14	1.24	0.56	–	−0.83	–	0.49	–	0.69	–
**Service access**										
Both NHS and non-NHS										
NHS only	−2.03	–	−1.27	–	−0.60	–	−2.97	–	−1.15	–
Non-NHS only	−0.74	–	−1.16	–	−1.17	–	−1.17	–	−0.32	–
No services accessed	−0.08	–	−0.49	–	−1.93	–	−0.63	–	−0.61	–

Logistic regressions are univariate with adjustment for allocation; baseline was also adjusted in models of injury thoughts and NSSI.

Values shown are non-exponentiated coefficients (logit scale); coefficients shrunk to zero in adaptive LASSO models are shown as (−).

Gestures and attempts were not modelled due to low frequency of occurrence.

The SITBI also asked participants how likely they were to have thought/behaviour in the future. Mean likelihood scores for each domain at each time point are shown in Supplementary File S3, Supplementary Table S2.

The SBQ-ASC measures suicidal thoughts and behaviours, with higher scores indicating more thoughts/behaviours. At baseline, the mean score in the AASP + usual care arm was 16.48 (SD 2.77) and the mean score in the usual care arm was 15.05 (SD 3.17) (Supplementary File S3, Supplementary Fig. S2). Response to a singular question on the SBQ-ASC, ‘When you experience intense thoughts about ending your life, how likely are you to act on them?’, was also explored separately in a pre-specified analysis. Mean responses to this question at baseline and 6 months, respectively, were 2.95 (SD 1.95) and 2.95 (SD 1.22) in the AASP arm and 2.88 (SD 1.12) and 3.00 (SD 1.27) in the usual care arm.

Descriptive statistics and analysis of the VEQ, in which higher scores correspond to more negative life experiences in ten different domains and summed overall, are shown in Supplementary File S3, Supplementary Table S1.

Table 2 shows the results of applying the adaptive LASSO method, with univariate logistic regression for comparison, to explore the associations between baseline sociodemographic variables and binary SITBI outcomes at six months. Broadly speaking, education level and employment status were most consistently retained in the adaptive LASSO models: compared with education to A-levels or below, education to a postgraduate level was associated with lower likelihood of ideation, NSSI, and any behaviour, while self-employment was associated with lower likelihood of suicide plans, NSSI, and any behaviour at six months. This suggests education level and/or employment status may be important stratification variables in future definitive trials.

All the participants in the study responded to the health economics questionnaire tools. The completion rates when measured as a proportion of all returned questionnaires were generally high (>85%) in both groups of participants. In a definitive trial data from the Resource Utilisation Questionnaire will be used to determine TAU. Completion rates were excellent for most items across all time point, however there were lower completion rates (∼42% in the comparator and ∼ 52% in the intervention at follow-up) for a question on medication details. It is reasonable to have lower completion rates for this question on medication details as it would generally be hard to recall the drug dose, start date or end date of all the medication the participants listed. However, the participants in this study were very committed to providing accurate data and so would rather not answer when they were not sure. The completion rates for time travel questionnaire (>79% in both groups), EQ-5D-5L (∼96%) and EQ-5D-VAS (∼87%) were also high. Overall, the health economics analysis showed that it would be feasible to collect data from autistic participants using the resource utilisation, time-travel questionnaire and EQ-5D-5L (including the EQ-5D-VAS) in a future definitive trial.

Forty-seven interviews were undertaken with participants after the completion of the six-month follow-up. All interviews included information regarding the research process (see Supplementary File S2). Participants generally found the research methods to be acceptable. The research process was seen as well-designed and accessible for autistic people due to the flexible approach which accommodated individual needs. However standardized measures needed less ambiguous language and space for open questions to capture the autistic experience of suicidality. Most participants experienced benefits from taking part in the research, including contributing to autism research, increased self-understanding, validation and being able to express themselves.

Participants in the AASP arm completed additional questions related to the Safety Plan. They reported a helpful process, receiving varying levels of support to identify and communicate their feelings, needs and experiences. They were motivated to use it by meaningful content and varied formats and suggested different versions, further prompts and navigation to increase usage.

In the AASP arm there were 3 Serious Adverse Events (SAEs) impacting 3 participants and there were 8 Events of Special Interest (ESIs) impacting 5 participants. In the control arm there were 9 SAEs impacting 5 participants and 16 ESIs impacting 11 participants. None were related to study participation.

Analysis of fidelity of delivery of the AASP was undertaken by an independent rater (IG). An additional independent rater (JR) met with IG to ensure inter-rater reliability using the fidelity checklist. 20% (n = 5) of AASP session recordings were assessed for therapeutic components and adherence to content. Fidelity ratings for delivery of the AASP were excellent overall: 94% for therapeutic components and 91% for adherence to content.

## Discussion

We report results from the first pilot randomised controlled trial exploring feasibility and acceptability of AASP. A majority (68%) of autistic participants were satisfied with the AASP, but fewer (41%) rated the AASP as useable. Participant feedback on the AASP and research methods was positive overall, with suggested adaptations to outcome measures to clarify language and better capture autistic experiences of self-harm and suicidality. There was excellent retention in both the AASP and control arms, and the majority of all study outcome measures were completed with few missing data points. Fidelity of delivery of AASP was excellent.

Results suggest that a definitive randomised controlled trial to determine the effectiveness and cost-effectiveness of AASPs is feasible and acceptable. However, results also suggest that some minor changes are needed to the outcome measures and to the AASP prior to a future definitive trial. For example, the SUS indicated that 41% of autistic participants rated the AASPs as useable. However, this is not surprising as the scale prioritises patients being able to complete the intervention independently and quickly, whereas feedback from autistic adults indicated that they required a flexible approach to completing AASP, with support of a trusted person to help identify warning signs and coping strategies, across more than one session if needed. Autistic adults also suggested adaptations to measures to make them clearer and more relevant to their unique experiences. These results are consistent with previous research, showing that outcome measures and interventions developed for the general population need to be adapted to better meet the needs of autistic people, including clearer language/instructions, more time for processing and to build rapport, and support to identify warning signs and support strategies.^13^^,^^21^^,^^28^^,^^29^

There is evidence that safety plans can reduce self-harm, suicidal thoughts and behaviours in a wide range of clinical groups,^10^ and safety plans have been recommended for use with autistic adults.^8^ Ours is the first study of AASP. Limitations include the small sample size below the initially intended sample of 70 participants due to difficulties with recruitment during the COVID-19 pandemic, although there is no single recommended sample size for a pilot or feasibility study, and the data gathered from the sample was sufficient to estimate the parameters of a future definitive trial. As autistic participants self-referred into the study from public adverts, data are not available regarding how many participants were approached with an invitation to take part in the study, compared to how many of these participants consented to take part. The study sample was not representative of the autism population generally. However, the high rates of low income, non-binary gender, female gender and late diagnosis of autism in adulthood may actually be representative of autistic people at higher risk of self-harm and suicide.^5^ Nevertheless, a future definitive trial will need to take further steps to ensure a more representative sample, particularly in terms of ethnicity. Autistic participants particularly favoured the flexible approach to the research and AASP, with one of the key adaptations of the opportunity of completing the plan with a trusted person across more than one session if needed.^13^ Such adaptations have been shown to improve effectiveness of other autism adapted interventions^28^^,^^29^ and will also likely be needed for the AASP.^13^ However, this does mean that AASPs may be more feasible to be conducted in settings with repeat appointments with patients (e.g., longer term psychological therapy), as opposed to more time pressured environments such as emergency departments. Additionally, clinicians in time pressured contexts may choose not to take up the AASP for the autistic people they support in favour of the original briefer version of the safety plan. Future research should explore clinician uptake of AASP in different clinical settings, and what key components of AASP could be prioritised in more time pressured clinical settings.

Despite autistic people being at high risk of self-harm and suicide, this study addressed a key research gap, namely that there are no suicide prevention interventions yet developed or evaluated in this group. Previous research has shown that outcome measures and interventions adapted for autistic adults improve the appropriateness and effectiveness of these tools.^21^^,^^28^^,^^29^ In England, the new government suicide prevention strategy^7^ prioritises adapted suicide prevention interventions such as safety plans developed with and for autistic people. The present findings from the first pilot randomised control trial of autism adapted safety plans suggests that a future definitive trial testing the efficacy of AASP is both feasible and acceptable. Future research should further explore the effectiveness and cost-effectiveness of AASP in routine clinical practice.

## Contributors

JR is the chief investigator who with SAC developed the study protocol alongside the other co-applicants (RC, ET, LV, SR, PH, EO, CW). JR and SC had overall responsibility for the management and delivery of the trial. IG was the researcher at University of Nottingham and JG and MP were the researchers at Newcastle University. IG and JG undertook data collection. MP and IG curated the data. Formal analysis was undertaken by JW, NB, IG and MP. The original draft of this paper was written by JR, SC, MP, JW, NB and IG. All authors reviewed the final submitted manuscript. JR, JW, NB, IG and MP accessed and verified the underlying data.

## Data sharing statement

Anonymised datasets used and/or analyses may be available from the corresponding author upon reasonable request.

## Declaration of interests

SC reports grants from the Economic and Social Research Council (ESRC) Autistica, Chief Scientist Office, the International Society for Autism Research and National Institute for Health Research (NIHR); occasional fees for delivering workshops and invited addresses; occasional fees for acting as an expert witness in coroners’ inquests; and participation in a trial data monitoring and ethics committee. RC is a Trustee and Science Council Member of MQ Mental Health Research, President of the International Association for Suicide Prevention, co-Chair of the Academic Advisory Group to the Scottish Government’s National Suicide Prevention Leadership Group, and a board member of the International Academy of Suicide Research. RC was a member of the National Institute for Health and Care Excellence’s guideline group for the management of self-harm; receives royalties from books, and occasional fees for workshops and invited addresses; and reports grants from the Medical Research Foundation, the Mindstep Foundation, Chief Scientist Office, Medical Research Council, Public Health Scotland, Scottish Government, NIHR, Shout 85258, Scottish Association for Mental Health, Zoetis Foundation, Jonathan’s Voice, ADHD UK, and the Barfil Charitable Trust. ET reports funding from the Garfield Weston Foundation, NIHR, American Foundation of Suicide Prevention, UKRI, MRC, Mental Health Research UK, Storm Skills. She has been a member of the International Covid-19 Suicide Prevention Research Collaboration Steering Group Member 2020- present and was on the Steering Committe for the National Confidential Inquiry into Suicide and Safety in Mental Health (NCISH) between 2019 and 2023. ET is past Trustee of the charity Collateral Global (until Oct 2023). The Self-Harm Research group led by ET were commissioned by Sussex NHS Trust Partnership to evaluate a school-based self-harm prevention programme in 2021. JR reports grants from Autistica, ESRC, University of Western Australia, Wellcome Trust; occasional honoraria for lectures/workshops at conferences/educational events; and participation in a trial steering committee as chair. MP reports grants from Edinburgh Mental Health Network, Autistic Centre for Excellence at Cambridge, Medical Research Foundation, Coventry University and ESRC Festival of Social Science; honoraria for invited talks received from National Autism Training Programme and Corporé case management. All other authors declare no competing interests.

## Appendix ASupplementary data

Supplementary File S3

AASP Study Protocol

Appendix 4 - Timepoints
