# Supplementary material for: Feasibility and acceptability of autism adapted safety plans: an external pilot randomised controlled trial
Source: eClinicalMedicine. 2024 Jun 1;73:102662. doi: 10.1016/j.eclinm.2024.102662 (PMC11165343; doi:10.1016/j.eclinm.2024.102662)
Supplement: Supplementary File S1 [file mmc1.docx]

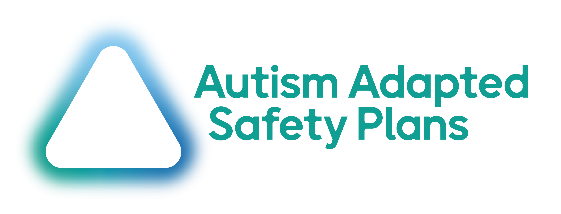


**Fidelity Ratings**

**Name of rater: ______________________________________________**

**Date AASP rated: ______________________________________________**

**Participant ID: ______________________________________________**

**Service provider name: ______________________________________________**

**Service provider organisation: ______________________________________________**

**Live rating Recorded rating**

## Fidelity to Delivery

**PLEASE INDICATE WHICH OF THE FOLLOWING COMPONENTS WERE COMPLETED IN THE AASP:**

**Please rate all items for each session, unless stated in brackets.**

|  | **To what extent was this used (please circle)** |
| --- | --- |
| **Session Structure** | N/A = not applicable  0 = not at all  1 = briefly covered but insufficiently  2 = covered adequately |
| 1. Objectives of AASP explained | N/A 0 1 2 |
| 1. Participant’s goals for AASP identified (e.g., targeting self-harm, suicidal thoughts, etc) | N/A 0 1 2 |
| 1. Objectives for AASP were summarised at the end by service provider | N/A 0 1 2 |
| 1. Service provider checked participant is happy with AASP/if they would like to amend anything |  |
| 1. Any questions were answered | N/A 0 1 2 |
| 1. Explanation of next steps for the study provided | N/A 0 1 2 |

| **Techniques used during session** | N/A = not applicable  0 = not at all  1 = minimal evidence  2 = several examples |
| --- | --- |
| 1. Collaborative approach.   I.e. The extent to which the AASP session is used in a way which allows reflection and discussion including sharing of experiences. | N/A 0 1 2 |
| 1. Non-blaming attitudes.   I.e. service provider demonstrates empathy and understanding of the participant’s position | N/A 0 1 2 |
| 1. Individualisation   I.e. service provider adjusts communication style and AASP to participant’s needs/preferences  Service provider encourage reflection on how the information relates to the participant. | N/A 0 1 2  N/A 0 1 2 |

| **Techniques used during session** | N/A = not applicable  0 = not at all  1 = minimal evidence  2 = several examples |
| --- | --- |
| 1. Exploration of sensory needs and ‘sticky’ thinking   E.g., acknowledging importance of sensory needs, collaborative discussion to workshop solutions to challenges | N/A 0 1 2 |
| 1. Checking for barriers   e.g., discussion of challenges relating to each step and collaboratively workshopping solutions where possible | N/A 0 1 2 |

| **Generic acceptable therapeutic components** | N/A = not applicable  0 = not at all  1 = minimal evidence  2 = several examples |
| --- | --- |
| 1. Service provider fosters an empathic, warm and genuine relationship with the participant | N/A 0 1 2 |
| 1. Service provider communicates effectively using appropriate empathic statements, reflections, clarification, verbal and non-verbal behaviours. | N/A 0 1 2 |
| 1. Emotional content is managed. i.e. a safe containing space is created | N/A 0 1 2 |
| 1. Service providers demonstrate flexibility in the delivery of the AASP for the specialist needs of the participant to optimise access and inclusion | N/A 0 1 2 |

| **Undesirable components** | N/A = not applicable  0 = not at all  1 = minimal evidence  2 = several examples |
| --- | --- |
| 1. Use of a purely didactic approach.   Didactic is defined by the lack of any opportunity for discussion, reflection and consideration of how information applies to the participant | N/A 0 1 2 |
| 1. Unnecessary deviation from the AASP/irrelevant content covered | N/A 0 1 2 |
| 1. Completely unstructured discussions | N/A 0 1 2 |
| 1. Not accepting participant’s expertise in their own life (e.g., including items in the AASP that participant does not think will be helpful) |  |
| 1. Other: Please describe | N/A 0 1 2 |

## Session 1 - Fidelity to Content

**PLEASE INDICATE WHICH OF THE FOLLOWING COMPONENTS WERE COMPLETED IN YOUR GROUP:**

| **AASP content** | N/A = not applicable  0 = not at all  1 = briefly covered but insufficiently  2 = covered adequately |
| --- | --- |
| - What is important to me? | N/A 0 1 2 |
| - Step 1 - What are my warning signs that I may start to have strong thoughts, feelings or urges to hurt myself and/or end my life? | N/A 0 1 2 |
| - Step 2 - What can I do to help distract myself? | N/A 0 1 2 |
| - Step 3 - People I can contact to ask for help | N/A 0 1 2 |
| - Step 4 - Professionals or agencies I can contact during a crisis | N/A 0 1 2 |
| - Step 5 - What can I do to make the environment around myself safer? | N/A 0 1 2 |
| - Step 6 - How can other people help support me? | N/A 0 1 2 |
| - Step 7 – Sharing my safety plan | N/A 0 1 2 |
| - Storing my safety plan | N/A 0 1 2 |
| **Optional: Resource Kit** | |
| - How do I feel? | N/A 0 1 2 |
| - Scales | N/A 0 1 2 |
| - Where can I go for help? | N/A 0 1 2 |
| - Self-care | N/A 0 1 2 |
| - Autism profile | N/A 0 1 2 |
| - Meltdown de-escalation strategies | N/A 0 1 2 |
| - Social support plan | N/A 0 1 2 |
