# Supplementary material for: Feasibility and acceptability of autism adapted safety plans: an external pilot randomised controlled trial
Source: eClinicalMedicine. 2024 Jun 1;73:102662. doi: 10.1016/j.eclinm.2024.102662 (PMC11165343; doi:10.1016/j.eclinm.2024.102662)
Supplement: Supplementary File S2 [file mmc2.docx]

**AASP**

**Feasibility Interview Questions**

**Autistic Person**

**Research Processes**

We are asking these questions to improve the way we might do a larger study in the future.

1. Was the information sheet helpful? Were the questions on the consent form clear and easy to answer?
2. Were any questions on the questionnaires difficult to talk about or answer?

Prompts:

- Which questions allowed you to best explain your experiences of self-harm and/or suicidality?
- Was the wording of any questions unclear?
- Do you think any questions were not relevant to the study?
- Was there anything else about suicide and / or self-harm we should have asked about? (e.g. impact of age, gender/sexuality, healthcare experiences, menopause)

1. Did it take a long time to do the questions or was the timing just right?
2. Is there anything else you would like to tell us about taking part in the research?
3. Were there any positive aspects of taking part in the research?
4. What are your thoughts on allocating people to safety plans and control arms? Prompts: What are your experiences of how this was done? What are your thoughts on whether we gave you enough information?

**AASP completion**

This version of the safety plan has been co-produced with autistic people but there is always room for improvement.

1. Were there any aspects of the safety plan that you found helpful?
2. What didn’t you like about the safety plan?

Prompts:

- What could we improve?
- Could it be adapted further for autistic people?

1. Is there anything that you would change about the format of the plan?

Prompts:

- Would the plan be better in a different format e.g. more visual, sensory, 3D objects?
- Did the online appointment(s) to develop the plan work for you?

1. Would you recommend AASPs to other autistic people?
