# Supplementary material for: Feasibility and acceptability of autism adapted safety plans: an external pilot randomised controlled trial
Source: eClinicalMedicine. 2024 Jun 1;73:102662. doi: 10.1016/j.eclinm.2024.102662 (PMC11165343; doi:10.1016/j.eclinm.2024.102662)

Supplementary Table 1. Summary and analysis of SITBI thoughts and behaviours

	AASP + usual care (n=25)				Usual care (n=24)				
	N	Yes n (%)	No n (%)	NA n (%)	N	Yes n (%)	No n (%)	NA n (%)	Odds ratio (95% CI)
**Ideation**									
Baseline	25	25 (100%)	0 (0%)	0 (0%)	24	23 (96%)	1 (4%)	0 (0%)	··
1 month	18	7 (39%)	3 (17%)	8 (44%)	24	13 (54%)	6 (25%)	5 (21%)	0·66 (0·09, 4·72)
6 months	23	10 (44%)	6 (26%)	7 (30%)	23	9 (39%)	7 (30%)	7 (30%)	1·69 (0·36, 8·78)
**Gestures**									
Baseline	25	4 (16%)	21 (84%)	0 (0%)	24	1 (4%)	23 (96%)	0 (0%)	··
1 month	18	2 (11%)	3 (17%)	13 (72%)	23	0 (0%)	1 (4%)	22 (96%)	0·27 (0·01, 2·55)
6 months	23	1 (4%)	4 (17%)	18 (78%)	23	0 (0%)	4 (17%)	19 (83%)	1·45 (0·22, 12·70)
**Plans**									
Baseline	25	22 (88%)	3 (12%)	0 (0%)	24	19 (79%)	5 (21%)	0 (0%)	··
1 month	18	3 (17%)	7 (39%)	8 (44%)	24	2 (8%)	12 (50%)	10 (42%)	2·22 (0·26, 22·70)
6 months	23	4 (17%)	14 (61%)	5 (22%)	23	1 (4%)	12 (52%)	10 (43%)	3·95 (0·47, 87·40)
**Attempts**[1]									
Baseline	25	23 (92%)	2 (8%)	0 (0%)	24	17 (71%)	7 (29%)	0 (0%)	··
1 month	18	1 (6%)	12 (67%)	5 (28%)	23	1 (4%)	17 (74%)	5 (22%)	··
6 months	23	0 (0%)	20 (87%)	3 (13%)	22	1 (5%)	18 (82%)	3 (14%)	··
**Thoughts of injury**									
Baseline	25	20 (80%)	5 (20%)	0 (0%)	24	20 (83%)	4 (17%)	0 (0%)	··
1 month	18	5 (28%)	6 (33%)	7 (39%)	23	8 (35%)	4 (17%)	11 (48%)	0·56 (0·07, 4·34)
6 months	23	7 (30%)	8 (35%)	8 (35%)	23	8 (35%)	6 (26%)	9 (39%)	1·01 (0·19, 5·72)
**Non-suicidal self-injury**									
Baseline	25	21 (84%)	3 (12%)	1 (4%)	24	18 (75%)	6 (25%)	0 (0%)	··
1 month	18	7 (39%)	7 (39%)	4 (22%)	23	5 (22%)	8 (35%)	10 (43%)	1·75 (0·35, 9·43)
6 months	23	6 (26%)	10 (44%)	7 (30%)	23	6 (26%)	9 (39%)	8 (35%)	0·59 (0·09, 3·37)
**Any behaviour**									
Baseline	25	25 (100%)	0 (0%)	··	24	24 (100%)	0 (0%)	··	··
1 month	18	11 (61%)	7 (39%)	··	23	16 (70%)	7 (30%)	··	0·50 (0·09, 2·37)
6 months	23	16 (70%)	7 (30%)	··	22	13 (59%)	9 (41%)	··	3·15 (0·82, 14·00)

Footnotes:
NA: not applicable or unable to answer

Ideation: ever had thoughts of killing oneself. Plan: made a plan to kill oneself. Gesture: done something to lead someone to believe one wanted to kill themselves when they really had no intention of doing so. Attempt: attempt to kill oneself with at least some intent to die; thoughts of injury: had thoughts of purposely hurting oneself without wanting to die. NSSI: engaged in NSSI; any behaviour refers to a response of ‘yes’ to at least one of the above thoughts/behaviours.

[1]Attempts could not be modelled due to low frequency.

Supplementary Table 2. Summary and analysis of likelihood scores for SITBI thoughts and behaviours

	AASP+usual care (n=25)		Usual care (n=24)		
	N	Mean (SD)	N	Mean (SD)	Mean difference (95% CI)
**Ideation**					
Baseline	22	3·77 (0·61)	20	3·60 (0·68)	··
1 month	17	3·41 (0·94)	21	3·57 (1·03)	-0·20 (-0·96, 0·56)
6 months	23	3·13 (1·36)	21	3·52 (0·68)	-0·44 (-1·20, 0·31)
**Gestures**					
Baseline	5	1·60 (1·52)	1	2·00 (NA)	··
1 month	9	2·11 (1·76)	9	0·44 (1·33)	··
6 months	5	1·80 (2·05)	5	0·80 (1·10)	··
**Plans**					
Baseline	20	3·20 (1·15)	15	2·10 (1·49)	··
1 month	14	2·71 (1·44)	15	2·73 (1·53)	-1·00 (-1·90, -0·18)
6 months	20	2·48 (1·43)	17	2·71 (1·36)	-1·00 (-1·80, -0·19)
**Attempts**					
Baseline	21	2·38 (1·20)	14	2·39 (1·39)	··
1 month	14	2·29 (1·59)	19	2·26 (1·59)	-0·35 (-1·10, 0·39)
6 months	21	2·19 (1·47)	18	1·89 (1·37)	-0·32 (-1·20, 0·53)
**Thoughts of injury**					
Baseline	18	3·06 (1·21)	16	2·88 (1·54)	··
1 month	15	3·20 (1·15)	19	2·63 (1·80)	0·22 (-0·55, 0·98)
6 months	18	2·78 (1·56)	16	3·06 (1·39)	-0·40 (-1·60, 0·78)
**Non-suicidal self-injury**					
Baseline	19	2·95 (1·43)	16	2·94 (1·65)	··
1 month	15	3·27 (1·03)	18	2·28 (1·78)	0·42 (-0·18, 1·00)
6 months	18	3·06 (1·30)	18	2·31 (1·67)	0·43 (-0·57, 1·40)

Footnote: Variation in N is due to exclusion of responses ‘not applicable’ or ‘unable to answer’

Supplementary Table 3. Description and analysis of VEQ scores across themes and in total.

	AASP+usual care (n=25)		Usual care (n=24)		
	N	Mean (SD)	N	Mean (SD)	Mean difference (95%CI)
**Education**					
Baseline	24	4·71 (2·54)	24	4·38 (1·95)	··
1 month	4	0·00 (0·00)	7	0·71 (1·11)	-0·34 (-4·20, 3·50)
6 months	5	0·40 (0·89)	11	1·82 (1·72)	-2·00 (-5·10, 1·10)
**Employment**					
Baseline	23	6·09 (4·43)	22	5·64 (2·95)	··
1 month	15	0·67 (1·18)	18	0·78 (1·22)	-0·01 (-0·91, 0·89)
6 months	15	7·33 (25·38)	16	1·50 (2·58)	9·20 (-5·50, 24·00)
**Finances**					
Baseline	25	2·36 (2·12)	24	2·13 (2·17)	··
1 month	18	1·22 (1·83)	24	0·50 (1·02)	0·64 (-0·05, 1·30)
6 months	20	1·45 (1·93)	23	0·83 (1·03)	0·47 (-0·28, 1·20)
**Social services**					
Baseline	7	2·00 (2·08)	5	1·40 (1·95)	··
1 month	5	0·20 (0·45)	5	0·00 (0·00)	0·33 (-1·70, 2·40)
6 months	3	0·00 (0·00)	6	0·33 (0·82)	··
**Criminal justice system**					
Baseline	25	0·56 (1·36)	23	0·65 (1·34)	··
1 month	17	0·12 (0·49)	24	0·00 (0·00)	0·09 (-0·12, 0·30)
6 months	21	0·00 (0·00)	19	0·11 (0·46)	-0·10 (-0·31, 0·12)
**Childhood victimisation**					
Baseline	25	13·48 (4·08)	24	12·92 (3·63)	··
**Adulthood victimisation**					
Baseline	25	8·12 (4·94)	24	7·63 (4·51)	··
1 month	17	1·41 (2·62)	24	0·92 (1·18)	0·80 (-0·28, 1·90)
6 months	20	1·60 (3·30)	23	1·65 (2·27)	0·23 (-1·30, 1·80)
**Domestic abuse**					
Baseline	20	4·00 (3·34)	21	3·14 (3·18)	··
1 month	5	0·80 (1·79)	12	0·00 (0·00)	0·73 (-0·72, 2·20)
6 months	6	0·83 (2·04)	10	0·00 (0·00)	0·38 (-2·10, 2·80)
**Mental illness**					
Baseline	25	9·12 (2·57)	24	8·38 (2·93)	··
1 month	17	4·00 (2·09)	24	3·42 (2·10)	0·56 (-0·79, 1·90)
6 months	21	4·19 (2·58)	23	3·70 (2·85)	0·39 (-1·1, 1·9)
**Social support**					
Baseline	24	3·92 (2·39)	24	4·08 (2·04)	··
1 month	17	4·53 (1·66)	24	4·67 (1·81)	0·04 (-1·10, 1·20)
6 months	20	5·00 (1·75)	22	5·09 (1·38)	0·05 (-0·92, 1·00)
**All themes**					

Baseline	25	51·28 (18·03)	24	48·33 (13·07)	··
1 month	18	11·56 (6·70)	24	10·29 (3·42)	1·50 (-1·20, 4·30)
6 months	22	16·64 (19·71)	24	12·58 (6·46)	4·6 (-3·8, 13·0)

Variation in N is due to exclusion of responses ‘not applicable’ or ‘unable to answer’

Supplementary Figure 1. Parallel line plot of change in likelihood of future suicide plans

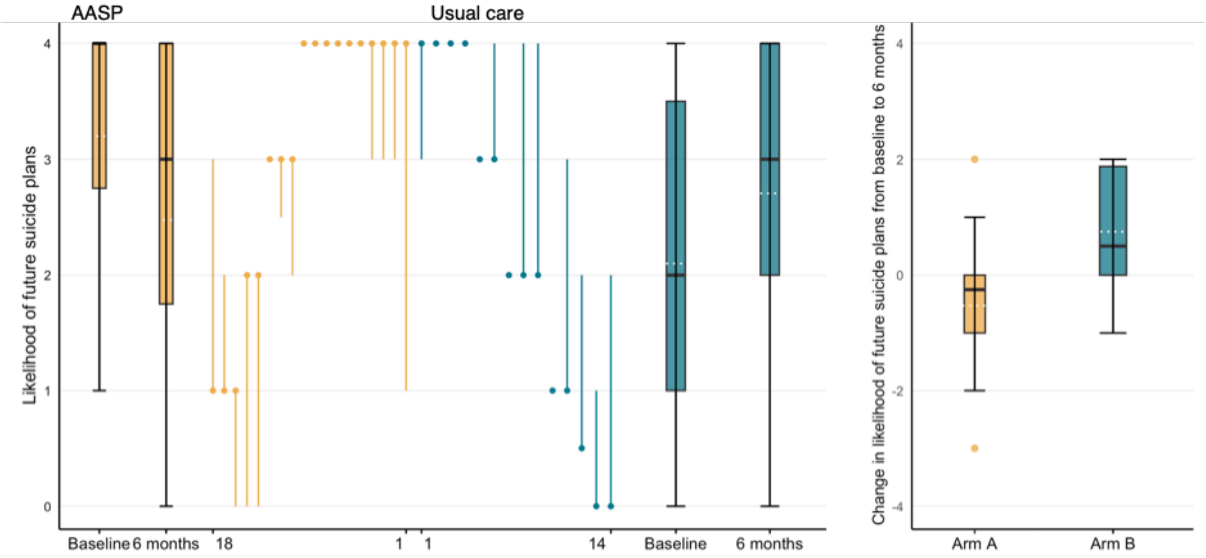

Figure footnote: The parallel line plot includes 1 vertical line for each participant which extends from the baseline value (point) to the 6-month value. Descending lines indicate an improvement; lack of lines indicate no change. Baseline values are arranged in ascending order for the AASP arm and descending order for the usual care arm. The ends of the boxes in the boxplots indicate the first and third quartiles; the middle black line indicates the median and the white dashed line indicates the mean. Whiskers extend to the upper and lower adjacent values, the location of the furthest point within a distance of 1.5 interquartile ranges from the first and third quartiles. Dots indicate extreme values. Likelihood is scored on a scale of 0 (low/little) to 4 (very much/severe).

Supplementary Figure 2. Parallel line plot showing change in SBQ-ASC score from baseline to 6 months

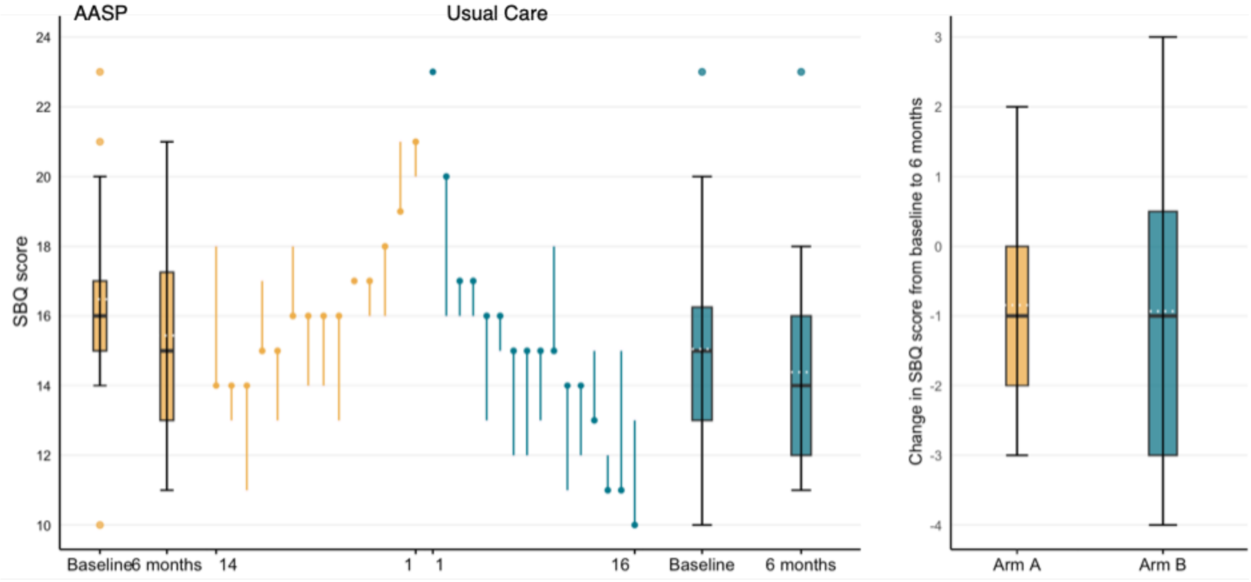

Supplement: Supplementary File S3 [file mmc3.pdf]
