# Supplementary material for: Feasibility and acceptability of autism adapted safety plans: an external pilot randomised controlled trial
Source: eClinicalMedicine. 2024 Jun 1;73:102662. doi: 10.1016/j.eclinm.2024.102662 (PMC11165343; doi:10.1016/j.eclinm.2024.102662)
Supplement: Appendix 4 - Timepoints [file mmc5.docx]

**Appendix 4. Pre-published time points for collection of outcome measures (taken from Rodgers et al., 2023)^12^**

| **Procedure** | **Screening** | **Baseline** | **1 Month Follow-up (F1)** | **6 Month Follow-up (F2)** |
| --- | --- | --- | --- | --- |
| **Autistic adults & professionals** |  |  |  |  |
| Eligibility checklist | X | .. | .. | .. |
| Informed consent | X | .. | .. | .. |
| Wellbeing plan (to note adaptations, participant safety, and emergency contact) | X | .. | .. | .. |
| **Autistic Adults Only** |  |  |  |  |
| Demographics^a^ | .. | X | .. | X |
| MINI | .. | X | .. | .. |
| SITBI | .. | X | X | X |
| VEQ^b^ | .. | X | X | X |
| SBQ-ASC | .. | X | .. | X |
| EQ-5D-5L | .. | X | .. | X |
| Resource Utilisation Questionnaire | .. | X | .. | X |
| Time and Travel Questionnaire | .. | X | .. | X |
| Randomisation^c^ | .. | X | .. | .. |
| Acceptability and feasibility semi-structured interview for autistic adults | .. | .. |  | X |
| SUS | .. | .. | .. | X^d^ |
| CSQ-8 | .. | .. | .. | X^d^ |
| **Professionals Only** |  |  |  |  |
| Acceptability and feasibility semi-structured interview for professionals | .. | .. | .. | X |

Footnotes:

^a^ Demographics to include – Socio-economic status, employment, housing, access to support, physical health, and education.

^b^ At baseline, this questionnaire asks about their entire life. At F1, it asks about the past month. At F2, it asks about the past 5 months.

^c^ Randomisation took place following completion of baseline assessment.

^d^ Only completed by participants allocated to the AASP arm.
